# Extracellular vesicle microRNA signature as a highly accurate diagnostic biomarker for human brucellosis

**DOI:** 10.3389/fcimb.2026.1806232

**Published:** 2026-05-22

**Authors:** Junwei Wu, Lei Zhang, Yingxin He, Haiyi Deng, Feiyang Cao, Yuheng Liu, Peiying Peng, Jin Su, Jie Wei, Xiaoye Lin, Qingxia Yi, Lei Zhan, Xiaomao Yin, Yi Yang, Yao Liao, Lifu Wang

**Affiliations:** 1 KingMed School of Laboratory Medicine, Guangzhou Medical University, Guangzhou, China; 2 Institute of Medical Laboratory Big Data & Economics, Guangzhou Medical University, Guangzhou, China; 3 Engineering Technology Research Center of Intelligent Diagnosis for Infectious Diseases in Guangdong Province, Guangzhou, China; 4 Guangzhou Key Laboratory for Clinical Rapid Diagnosis and Early Warning of Infectious Diseases, Guangzhou, China; 5 Medical Department, Guangdong Provincial Engineering Research Center for Early Warning and Diagnosis of Respiratory Infectious Diseases, Guangzhou, China; 6 School of Pharmaceutical Sciences, Guangzhou Kingmed Center for Clinical Laboratory, Guangzhou, China; 7 Medical Department, Xizang Minzu University, Xianyang, China; 8 The Second Affiliated Hospital, Guangzhou Medical University, Guangzhou, China; 9 School of Pharmaceutical Sciences, Southern Medical University, Guangzhou, China; 10 Department of Laboratory Medicine, Red Cross Hospital, Jinan University, Guangzhou, China

**Keywords:** *Brucella*, brucellosis, diagnosis, extracellular vesicles, microRNA

## Abstract

**Background:**

Brucellosis remains an important public health problem globally. Rapid and accurate diagnosis is crucial for the treatment of brucellosis. However, current diagnostic methods for brucellosis are limited, posing significant clinical challenges. Extracellular vesicles (EVs)-derived microRNAs (miRNAs) potentially offer a novel, non-invasive approach for accurate diagnosis of brucellosis.

**Methods:**

Small RNA sequencing was conducted to identify candidate miRNAs as potential diagnostic biomarkers in serum-derived EVs from patients with brucellosis and healthy individuals. These miRNA candidates were further validated in serum-derived EVs, serum free of EVs (EVs-free), and serum within an exploratory set and a preliminary validation set using a quantitative reverse transcription-polymerase chain reaction (qRT-PCR) and a logistic regression model to establish and validate the diagnostic signature.

**Results:**

The sequencing analysis initially screened and identified a panel of 52 overexpressed miRNAs in serum-derived EVs from patients with brucellosis. Subsequently, qRT-PCR demonstrated that 10 of the top 12 most differentially expressed miRNAs (miR-20a-5p, miR-320a-3p, let-7b-5p, miR-374a-5p, miR-93-5p, miR-186-5p, let-7d-5p, miR-151a-3p, miR-361-5p, and miR-98-5p), effectively distinguished patients with brucellosis from healthy individuals. Among single miRNAs, miR-361-5p (area under the curve [AUC], 0.917 [95% CI, 0.760 -1.000]) has the largest AUC. After logistic regression analysis and qRT-PCR, it was found that a miRNA signature (miR-20a-5p and miR-93-5p) in serum-derived EVs performed more robust in differentiating between patients with brucellosis and healthy individuals (exploratory set: AUC = 1.000; preliminary validation set: AUC = 0.988, 95% CI: 0.949 -1.000). Moreover, there was no significant difference in the expression levels of this miRNA signature in EVs-free and serum. EVs were enriched with miRNAs that are closely associated with *Brucella* infection.

**Conclusion:**

MiR-20a-5p and miR-93-5p in serum-derived EVs serve as highly accurate diagnostic biomarkers for brucellosis, offering a rapid and reliable alternative to conventional diagnostic methods.

## Introduction

Brucellosis, one of the most common zoonotic diseases worldwide, is caused by various species of the bacterial genus *Brucella* (World Health Organization, [[NoYear]]a). Among *Brucella* species, *Brucella melitensis*, *Brucella abortus*, and *Brucella suis* exhibit high virulence in their natural hosts and are also pathogenic to humans (Jiang et al., 2013; Roop et al., 2021). Brucellosis is an important human disease in many parts of the world especially in the Mediterranean countries of Europe, north and east Africa, the Middle East, south and central Asia and Central and South America and yet it is often unrecognized and frequently goes unreported. Brucellosis typically causes flu-like symptoms, including fever, weakness, malaise and weight loss. However, the disease may present in many atypical forms. Many patients have mild symptoms, which can easily lead to missed diagnoses or misdiagnosis (World Health Organization, [[NoYear]]a; World Health Organization, [[NoYear]]b). Globally, around 3.5 billion people live in brucellosis-endemic regions, with an estimated 2.1 million new human infections each year (Rossetti et al., 2017; Laine et al., 2023). In the past ten years, the global incidence and distribution of brucellosis have changed significantly in many countries. Countries with high incidence rates include Kenya (203.07 cases per 100,000 population), Yemen (89.96), Syria (47.26), Greece (42.96), and Eritrea (21.82) (Wang and Jiang, 2020). In Ningxia, China, from 2010 to 2024, brucellosis cases were reported in 22 counties, with a total of 35,665 confirmed cases (Duan et al., 2026). Although *Brucella* infection is a significant issue for livestock and is recognized as a zoonotic disease that has adverse effects on the reproductive system, potentially leading to infertility, there is limited knowledge and understanding of human brucellosis (Franco et al., 2007; Roop et al., 2021). Brucellosis is a systemic febrile disease; owing to its diverse clinical spectrum, it is also considered a non-specific influenza-like disease (Bosilkovski et al., 2010; Franc et al., 2018). Additionally, brucellosis can present clinical symptoms similar to those of other diseases (Barutta et al., 2013; Erdem et al., 2015; Zhang et al., 2021). Enzyme-linked immunosorbent assay (ELISA) is the index test most commonly used to evaluate brucellosis, followed by standard tube agglutination test, polymerase chain reaction (PCR), and Rose Bengal Test (Freire et al., 2024). These existing diagnostic methods for brucellosis have notable shortcomings, such as the long time required for bacterial culture, the lack of objectivity in serological tests, and the limited genomic targets for nucleic acid amplification assays (Yagupsky et al., 2019). Therefore, there is an urgent need for rapid and reliable methods for diagnosis and differential diagnosis of brucellosis.

Extracellular vesicles (EVs) are small membrane-bound vesicles secreted by all cells, and play an important role in cell-to-cell communication. Their cargos represent the pathophysiological status of the parent cells (Zaborowski et al., 2015; Bebelman et al., 2018). EVs are present in all biofluids, including blood, and serve as a transport system for various molecular components of the cells that they originate from. EVs thus enrich various molecular components in serum, including microRNA (miRNA) (Villarroya-Beltri et al., 2013; Matsumura et al., 2015). Previous studies have found that miRNAs can serve as diagnostic biomarkers for various diseases (He et al., 2021; Li et al., 2024). Encapsulated within a phospholipid bilayer membrane, EVs-miRNA is effectively protected from RNase degradation in body fluids, conferring high stability for diagnostic purposes. As signaling molecules actively packaged and secreted by cells under specific pathological conditions, EVs-miRNAs may be closely associated with disease and possess a certain degree of disease specificity (Mori et al., 2019). Moreover, the expression level of miRNAs has also been found to be significantly changed in brucellosis, and are known to play a key role in the pathogenesis and progression of the disease (Zheng et al., 2012; Budak et al., 2016b; Lecchi et al., 2019). Previous studies have shown that the downregulation of serum EVs-derived miR-let-7e-5p in patients with brucellosis promotes macrophage polarization toward the M2 phenotype (Li et al., 2025). In addition, *Brucella* can inhibit STING expression through miR-24, thereby disrupting the cytoplasmic surveillance mechanism and facilitating chronic infection (Khan et al., 2020). Another study has demonstrated that miR-21a-5p regulates the expression of interleukin-10 in macrophages, thereby affecting the survival of *Brucella* within host cells (Corsetti et al., 2018). Beyond the aforementioned mechanisms, the serum miRNA profile of patients can also be used for the differential diagnosis of brucellosis and pulmonary tuberculosis (Liang et al., 2023). EVs-miRNA detection is based on PCR technology and can yield results within hours after sample receipt, whereas traditional bacteriological testing often takes several weeks. Thus, EVs-miRNA detection offers significant advantages in enabling early diagnosis, timely treatment, and avoidance of unnecessary isolation. Besides, aberrant expression of EVs-miRNAs often occurs before serological antibodies appear; therefore, this approach may help address the limitations of false positives and false negatives associated with serological testing. Accordingly, miRNAs derived from EVs in serum have great potential as diagnostic biomarkers for brucellosis.

In the present study, we conducted a comparative analysis of serum EVs-miRNA profiles between patients with brucellosis and healthy individuals to identify a novel miRNA diagnostic signature for the diagnosis of brucellosis. We subsequently conducted a rigorous evaluation and validation of the miRNA signatures within clinical sets to determine their diagnostic performance for brucellosis. Our findings revealed that miRNAs were enriched in EVs derived from serum, and the miRNA profile in EVs differed significantly between patients with brucellosis and healthy individuals.

## Materials and methods

### Patient enrollment and study design

The serum samples of patients with brucellosis and healthy individuals used in this study were collected from tertiary Grade-A hospitals in Guangdong, China. This study included two groups, namely, the brucellosis subjects and the healthy subjects. According to the diagnostic criteria of the National Health Commission of the People’s Republic of China (NHC), positivity for the Rose Bengal Test as well as either the Tube Agglutination Tests, Brucellosis IgG Antibody, or IgM Antibody Test confirms a diagnosis of brucellosis (NHC, [[NoYear]]). All patients in the brucellosis subjects met the above diagnostic criteria. The healthy subjects consisted of healthy individuals who showed no signs of infection, cancer, or congenital diseases. A total of 48 human serum samples (from 24 patients in the brucellosis patients and 24 healthy individuals in the healthy subjects) were collected after obtaining informed consent. The samples were then randomly allocated to the exploratory set and preliminary validation set. The basic information for the samples is shown in **Table 1**.

**Table 1:** Details of participants.

Variables	Exploratory set		Preliminary validation set	
	Healthy subjects (n=12)	Brucellosis subjects (n=12)	Healthy subjects (n=12)	Brucellosis subjects (n=12)
Age (mean ± SD)	46 ± 5.58	46 ± 15.79	50 ± 4.95	55 ± 3.8
Gender				
Male, n (%)	5(41.7)	8(66.7)	7(58.3)	7(58.3)
Female, n (%)	7(58.3)	4(33.3)	5(41.7)	5(41.7)
Rose Bengal Test				
Positive, n (%)	0	12(100)	0	12(100)
Negative, n (%)	12(100)	0	12(100)	0
Tube Agglutination Test				
Positive (++++), n (%)	0	9(75)	0	11(91.7)
Positive (+++), n (%)	0	2(16.7)	0	0
Positive (++), n (%)	0	1(8.3)	0	1(8.3)
Positive (+), n (%)	0	0	0	0
Negative, n (%)	12(100)	0	12(100)	0
Brucellosis IgG				
Positive (+), n (%)	0	8(66.7)	0	12(100)
Negative, n (%)	12(100)	4(33.3)	12(100)	0
Brucellosis IgM	-		-	
Positive (+), n (%)	0	12(100)	0	11(100)
Negative, n (%)	12(100)	0	12(100)	0
No test, n	0	0	0	1
C-reactive Protein (mg/L, mean ± SD)	12.46 ± 12.7	45.21 ± 64.52	10.01 ± 7.4	41.12 ± 34.11
Procalcitonin (μg/L, mean ± SD)	0.17 ± 0.17	0.71 ± 1.41	0.46 ± 0.51	0.79 ± 1.33
White Blood Cell Count (109/L, mean ± SD)	6.75 ± 1.15	5.68 ± 2.69	5.65 ± 0.91	6.65 ± 2.82
Neutrophil Percentage (%, mean ± SD)	47.83 ± 4.19	59.59 ± 17.45	46.8 ± 2.24	59.8 ± 17.78
Clinical signs and symptoms				
Fever, n (%)	0	12(100)	0	12(100)
with Arthralgia, n (%)	0	4(33.3)	0	5(41.7)
with Low back pain, n (%)	0	4(33.3)	0	4(33.3)
Negative, n (%)	12(100)		12(100)	
Microbiological Test				
Positive (+), n (%)		3(25)		2(16.7)
Negative, n (%)		2(16.7)		2(16.7)
No test, n (%)	12(100)	7(58.3)	12(100)	8(66.7)
Final Diagnosis				
Brucellosis, n (%)		12(100)		12(100)
Negative, n (%)	12(100)		12(100)	

### EVs isolation

EVs were isolated from serum samples as previously described (Wang et al., 2022). Briefly, each serum sample was centrifuged at 300 ×g for 10 min using 1 ml at 4 °C to remove large debris, and the resulting supernatant was further centrifuged at 2000 ×g for 10 min and 10,000 ×g for 60 min at 4 °C to completely eliminate cellular debris, using a fixed angle rotor (Angle was 45 degrees, model #3331, D-37520 Refrigerated Centrifuge, Thermo Electron Corporation, USA). The resulting supernatant from each sample in the same group was then transferred to a Quick-Seal Centrifuge tube (Beckman Coulter, USA) and subsequently ultracentrifuged at 120,000 g for 90 min at 4 °C using an Optima L-100xp tabletop ultracentrifuge (Swinging bucket rotor, model SW40 Ti, Optima L-100xp, Beckman Coulter, USA). The supernatant was collected as an EVs-free preparation (EVs-free) to evaluate the potential contribution of residual extra-vesicular RNA, and the resultant pellet (EVs) was washed with phosphate-buffered saline (PBS), ultracentrifuged again, and re-suspended in PBS. Isolated EVs were quantified using a standard protein assay (Enhanced BCA Protein Assay Kit, Beyotime Biotechnology) and stored at − 80 °C.

### Identification of EVs

To identify the isolated EVs, negative-staining transmission electron microscopy (TEM) was used to analyze the characteristics of EVs. Briefly, EVs suspension was placed on a copper grid and negatively stained with 3% (w/v) aqueous phosphotungstic acid solution for 1 min. Then, the grid was examined with a FEI Tecnai G2 Sprit Twin TEM (FEI, USA). Expression of surface markers on EVs was detected by western blot analysis (WB) using antibodies against the positive markers CD9 (ab236630, Abcam) and CD63 (ab134045, Abcam), as well as the negative markers calnexin (66903, proteintech) and H3 (68345, proteintech), as primary antibodies, followed by treatment with a horseradish peroxidase-conjugated secondary antibody. Additionally, the EVs particles diluted with PBS were analyzed using Nanoparticle tracking analysis (NTA, NanoSight NS300, Malvern Instruments, United Kingdom).

### RNA extraction

RNA extraction was carried out under stringent RNase-free conditions. Total RNA was extracted from EVs using TRIzol^®^ Reagent in accordance with the manufacturer’s instructions. Then RNA quality was determined using a 5300 Bioanalyser (Agilent) and quantified using a Nanodrop ND-2000 spectrophotometer (NanoDrop Technologies). Only high-quality RNA samples (OD260/280 = 1.8~2.2, OD260/230 ≥ 2.0, RQN ≥ 6.5, 28S:18S ≥ 1.0, > 1μg) were used to construct the sequencing library.

### Library preparation and sequencing

RNA purification, reverse transcription, library construction, and sequencing were performed at Shanghai Majorbio Bio-pharm Biotechnology Co., Ltd. (Shanghai, China) according to the manufacturer’s instructions (Illumina, San Diego, CA). One microgram of total RNA per sample was used as input material for the small RNA library. Sequencing libraries were generated using the QIAseq miRNA Library Kit (Qiagen), following the manufacturer’s recommendations. The activated 5′ and 3′ adaptors were ligated to the total RNA. Then, the adaptor-ligated RNA was transcribed into first-strand cDNA by using reverse transcriptase and a random primer. A PCR reaction was performed using complementary primers, for 11–12 cycles, and fragments of appropriate size were isolated with a 6% Novex TBE PAGE gel. After quantification with Qubit 4.0, the single-end RNA-seq sequencing library was sequenced with the Illumina NovaSeq Xplussequencer.

### Quality control and reads mapping

Raw data (raw reads) of fastq format were first processed through fastp (Chen et al., 2018) with default parameters. After this step, clean data (clean reads) were obtained by removing 3′ end adapter, reads containing poly-N, low-quality bases (Sanger base quality of < 20) of the 3′ end, and sequencing adapters from raw data with the fastx toolkit software. All identical sequences of sizes ranging from 18 to 32 nt were counted and eliminated from the initial data set. Bowtie (Langmead and Salzberg, 2012) was used to annotate the chromosomal location against the reference genome data.

### Identification of miRNAs and differential expression analysis

Mapped small RNA tags were used to identify known miRNAs using the miRBase2 2.0 database (http://www.mirbase.org/) as reference. Then, the rest of tags were aligned against the Rfam database and Repbase database to remove ribosomal RNA (rRNA), transfer RNA (tRNA), small nuclear RNA (snRNA), small nucleolar RNA (snoRNA), and other ncRNA and repeats. The unannotated tags were predicted and identified as novel miRNAs using mirdeep2 (Friedländer et al., 2012) software, according to the tag positions in the genome and their hairpin structures. The expression level of each miRNA was calculated according to the transcripts per million reads (TPM) method. Differential expression analysis was performed using the DESeq2 (Love et al., 2014) or DEGseq (Wang et al., 2010). Differentially expressed genes (DEGs) with |log2FC| ≥ 1 and FDR ≤ 0.05(DESeq2) or FDR ≤ 0.001(DEGseq) were considered to be significantly differentially expressed.

### miRNA target gene predictions

Predictions of the target genes of miRNAs were performed with miRanda (Enright et al., 2003). The predicted target genes were annotated using the GO (http://www.geneontology.org/) and KEGG (http://www.genome.jp/kegg/) databases. Functional enrichment analysis, including GO and KEGG analyses, were performed to identify the targets that were significantly enriched in GO terms and metabolic pathways at P-adjust ≤ 0.05 compared with the whole-ref genes background. GO functional enrichment and KEGG pathway analyses were carried out using Goatools and KOBAS (Xie et al., 2011), respectively.

### Quantitative real-time reverse transcription-polymerase chain reaction

Expression of miRNAs was quantified using qRT-PCR. In brief, the extracted RNA was quantified using the ND-2000 spectrophotometer (NanoDrop Technologies). Complementary DNA (cDNA) was synthesized using oligo (dT) primers and a Thermo Scientific RevertAid First Strand cDNA synthesis kit (Thermo Scientific), according to the manufacturer’s protocol. This kit employs a poly(A) tailing method for miRNA first-strand cDNA synthesis, in which a poly(A) tail is added to the 3’ end of miRNAs and then reverse transcribed using an oligo(dT) primer, ensuring specificity of miRNA amplification. The expression of miRNAs was analyzed using a SYBR Green Master Mix kit (Takara, Japan) with the primers listed in **Supplementary Table 1**. The preset threshold for amplification was 40 cycles; samples that were not detected within 40 cycles could not be reliably quantified and were therefore excluded. In addition, U6 snRNA was used as internal control, and the fold-change in expression was calculated according to the 2^−ΔΔCT^ method.

### Statistical analysis

Statistical analyses and graphical representations were performed using SPSS version 25.0 (IBM Corporation, Armonk, NY, USA), R (version 4.0.3, https://cran.r-project.org/) and GraphPad Prism version 8.0 (GraphPad Software Inc., CA, USA). The two-tailed unpaired Student’s t-test was used to compare continuous variables in two groups. Multivariate logistic regression analysis with a stepwise backward selection method was performed for diagnostic modeling of screened and confirmed variables in the exploratory set. The predictive performance of the miRNA signatures was evaluated by calculating the area under the receiver operating characteristic (ROC) curves (AUC). Diagnostic cutoff values were optimized using the Youden index to maximize sensitivity and specificity. The calibration accuracy of the miRNA signatures was assessed through calibration plots, with internal validation performed using 1,000 bootstrap resampling iterations. A two-tailed *P*-value < 0.05 was considered to indicate statistical significance.

## Result

### Participants and clinical characteristics

A total of 48 participants were randomly enrolled into two independent sets, namely, the exploratory set and preliminary validation set. The exploratory set comprised 24 participants: 12 patients with brucellosis [age (mean ± Standard Deviations), (46 ± 15.79); 8 male (66.7%); 4 female (33.3%)] and 12 healthy individuals [(46 ± 5.88); 5 male (41.7%); 7 female (58.3%)]. The preliminary validation set consisted of 24 human serum samples from 12 patients with brucellosis [age, (55 ± 3.8); 7 male (58.3%); 5 female (41.7%)] and 12 healthy individuals [age, (50 ± 4.95); 7 male (58.3%); 5 female (41.7%)]. Compared with healthy controls, brucellosis patients exhibited elevated C-reactive protein (CRP) levels and a higher neutrophil percentage. The most common clinical symptom was fever, frequently accompanied by arthralgia or low back pain. Detailed participant information and laboratory diagnostic results are provided in **Table 1**.

### Differences in characterization and miRNA profiling of serum-derived EVs between patients with brucellosis and healthy individuals

**Figure 1:**
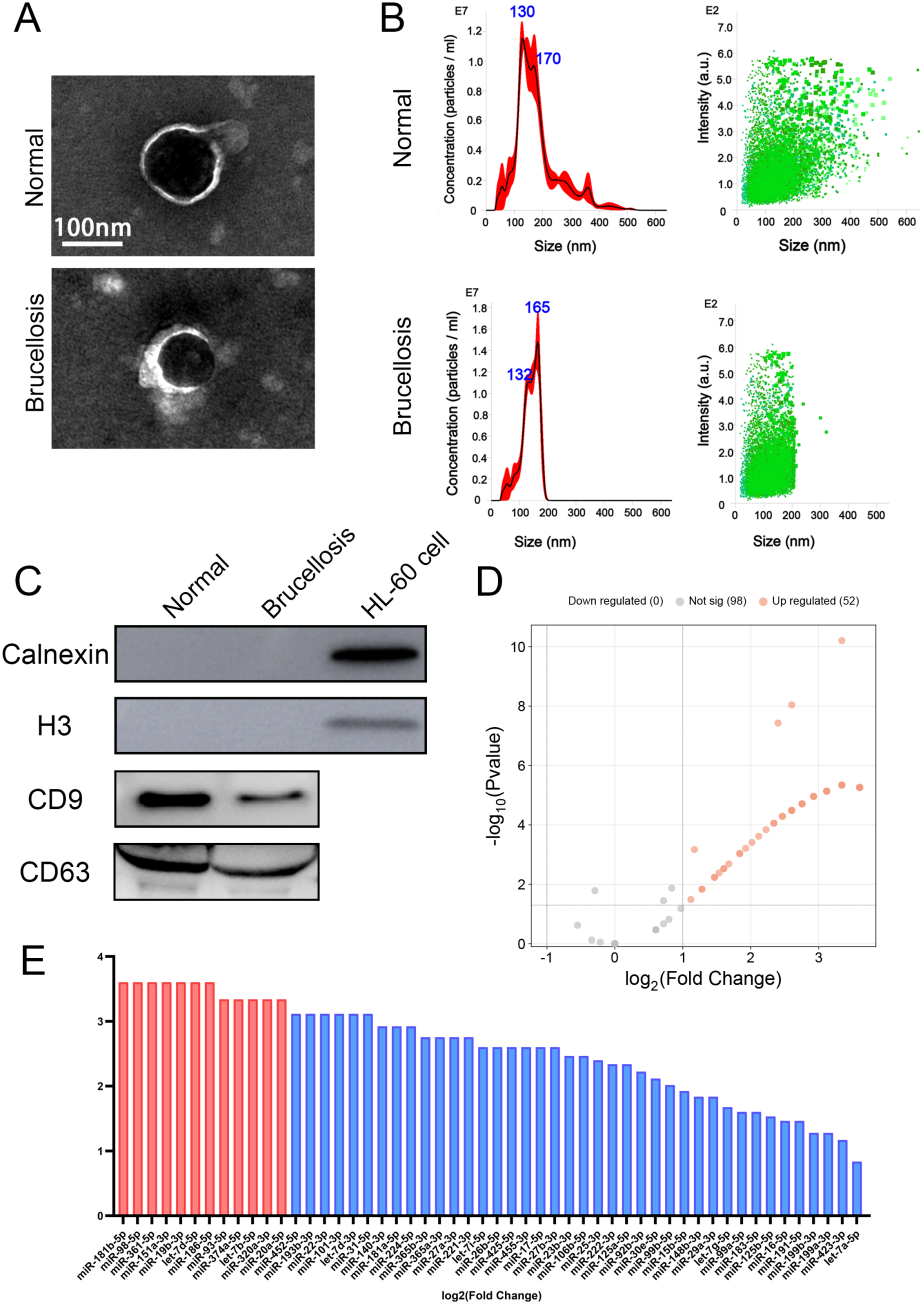
Characterization of serum-derived extracellular vesicles and significant differences in miRNA profiling in small RNA sequencing results between brucellosis patients (brucellosis) and healthy individuals (normal). **(A)** Representative transmission electron microscopy (TEM) images of serum-derived EVs. **(B)** Size distribution and concentration of EVs were investigated by Nanoparticle Tracking Analyses (NTA). **(C)** Expression of EVs surface markers (CD9 and CD63) was verified by western blot analysis (WB). **(D)** Volcano plots showing the distribution of 2-fold up-regulated miRNAs (*p* < 0.05, grey: no statistical significance, orange: Fold Change > 2.0). **(E)** Bar chart of 52 significantly up-regulated miRNAs (*p* < 0.05).

To investigate the characterization of serum-derived EVs in patients with brucellosis and healthy individuals, TEM was performed, and results confirmed the typical disc-shaped morphology of EVs (**Figure 1A**). The size distribution profile of the EVs was examined by NTA, revealing peak sizes of 130 nm for EVs derived from healthy individuals (normal-derived EVs [N-EVs]) and 165 nm for EVs derived from patients with brucellosis (brucellosis-derived EVs [B-EVs]). The sizes were found to range between 50 and 500 nm (N-EVs), and 50 and 200 nm (B-EVs), in diameter (**Figure 1B**). To achieve a reliable assessment of EVs preparations, we further performed BCA protein assay on the corresponding samples. The particle-to-protein ratio (particles per mL divided by protein mg per mL) was calculated for each sample. Notably, this ratio was significantly higher in EVs preparations than in the corresponding EVs-free fractions, for both healthy subjects and patients with brucellosis (**Supplementary Figure 1**). These results demonstrate that our ultracentrifugation-based EVs isolation protocol yields high-quality EVs preparations. Our findings suggest that *Brucella* infection may induce host cell activation or stress responses, thereby altering the composition and abundance of EVs cargo, which in turn promotes the release of larger vesicles. The size distribution of EVs has been widely documented to correlate with disease states (Carney et al., 2025; Su et al., 2025). Nevertheless, the precise mechanisms underlying the observed differences in EV size in the context of brucellosis remain to be fully elucidated. In addition, the expression of the EVs surface markers CD9 and CD63 was confirmed by western blot analysis. To further assess the purity of EVs preparations and rule out potential contamination from intracellular compartments, we also examined the negative EVs markers calnexin (an endoplasmic reticulum protein) and H3 (a nuclear protein) with positive control (HL-60 cell). Both calnexin and H3 were undetectable in EVs preparations, indicating the absence of contamination from cellular membranes or nuclear debris (**Figure 1C**; **Supplementary Figure 2**).

After combining the sera of individual samples from the same group and isolating EVs, we extracted total RNA and performed small RNA sequencing. Based on the sequencing result shown in the volcano plot, 150 miRNAs were identified, of which 52 miRNAs exhibited significant upregulation in B-EVs relative to N-EVs (**Figure 1D**; **Supplementary Table 2**). The bar chart revealed 52 miRNAs with at least 2-fold significant (Fold Change > 2.0 and *p* < 0.05) differences in expression (**Figure 1E**).

### Verification of EVs-derived miRNA profiles and construction of models in the exploratory set

**Figure 2:**
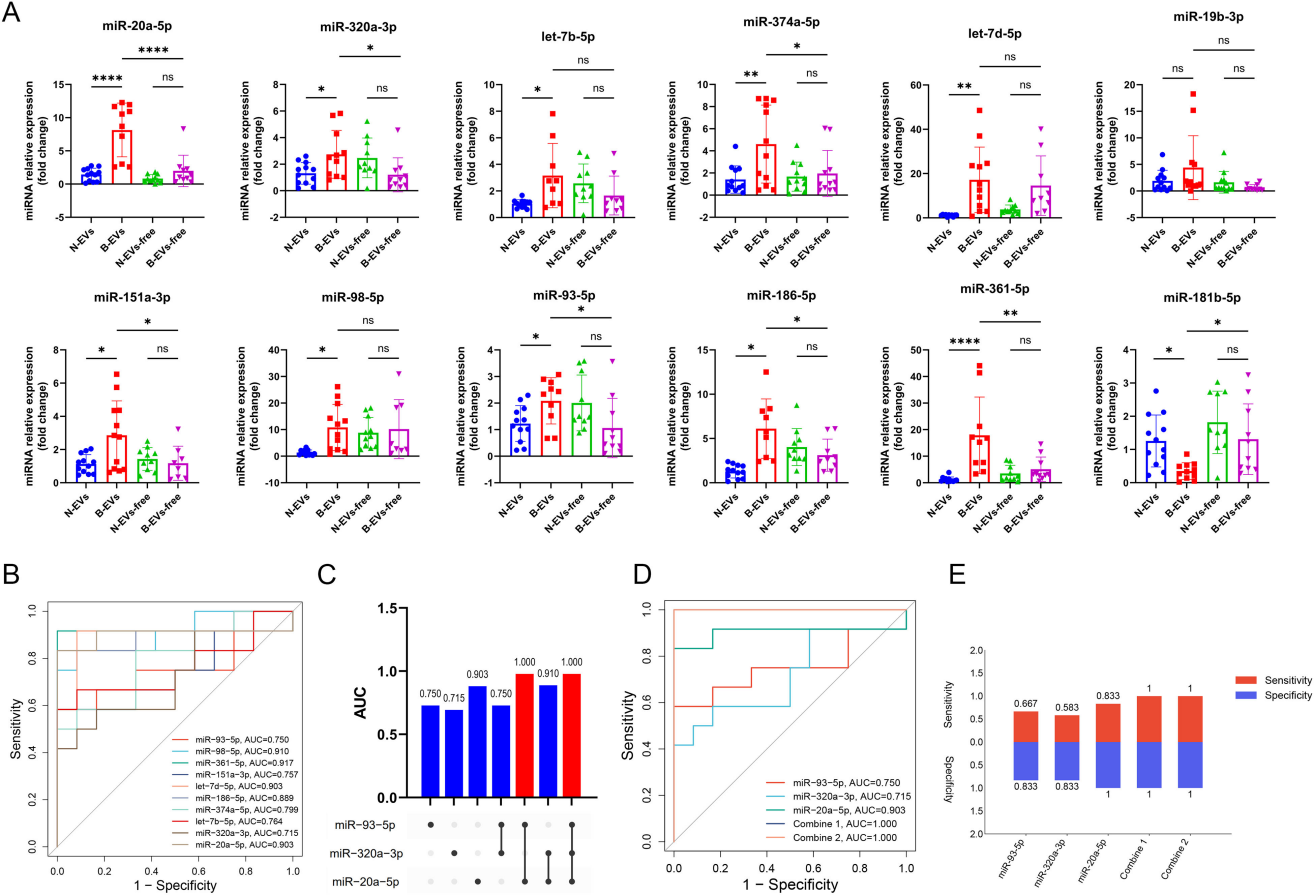
Quantitative real-time reverse transcription-polymerase chain reaction of miRNAs derived from the exploratory set, logistic regression analysis and comparison of the performance of diagnostic models. **(A)** Expression levels of miR-20a-5p, miR-320a-3p, let-7b-5p, miR-374a-5p, miR-93-5p, miR-186-5p, let-7d-5p, miR-19b-3p, miR-151a-3p, miR-361-5p, miR-98-5p, miR-181b-5p extracted from EVs and EVs-free (n=8-12). **(B)** Receiver operating characteristics (ROC) and Area under the ROC curve (AUC) of miR-20a-5p, miR-320a-3p, let-7b-5p, miR-374a-5p, miR-93-5p, miR-186-5p, let-7d-5p, miR-151a-3p, miR-361-5p, miR-98-5p extracted from EVs. **(C)** AUC, **(D)** ROC, **(E)** sensitivity and specificity of individual miRNAs, Combine 1 (miR-20a-5p + miR-320a-3p + miR-93-5p) and Combine 2 (miR-20a-5p + miR-93-5p). Data are mean ± SDs; * p < 0.05; ** p < 0.01; *** p < 0.001; **** p < 0.0001.

To validate the differential expression levels of EVs-miRNAs identified by sequencing, we selected the top 12 miRNAs with the highest fold change (**Figure 1E**) and performed qRT-PCR in the exploratory set (12 patients with brucellosis and 12 healthy individuals). Findings showed that the expression levels of miR-20a-5p, miR-320a-3p, let-7b-5p, miR-374a-5p, miR-93-5p, miR-186-5p, let-7d-5p, miR-151a-3p, miR-361-5p, miR-98-5p were significantly up-regulated in B-EVs relative to N-EVs (**Figure 2A**), consistent with the sequencing results. Comparisons of the 12 miRNAs between healthy individuals and patients with brucellosis showed no significant differences in either EV-free serum or serum (**Figure 2A**; **Supplementary Figure 3**). Moreover, the levels of these miRNAs were found to be higher in serum EVs from brucellosis patients than in EV-free serum, with the differences for several miRNAs (miR-20a-5p, miR-320a-3p, miR-374a-5p, miR-151a-3p, miR-93-5p, miR-186-5p, miR-361-5p and miR-181b-5p) being statistically significant (**Figure 2A**). These findings potentially indicate that disease-related miRNAs are more enriched in EVs in disease states (Zhou et al., 2021; Kim et al., 2023; Miyazaki et al., 2023). Furthermore, we selected 10 verified miRNAs with significant upregulation for ROC and AUC, evaluating their diagnostic performance individually. miR-20a-5p (AUC, 0.903 [95% confidence intervals (CIs), 0.745 to 1.000]), let-7d-5p (AUC, 0.903 [95%CIs, 0.761 to 1.000]), miR-361-5p (AUC, 0.917 [95%CIs, 0.760 to 1.000]), and miR-98-5p (AUC, 0.910 [95%CIs, 0.788 to 1.000]) showed excellent diagnostic performance, followed by miR-320a-3p (AUC, 0.715 [95%CIs, 0.501 to 0.929]), let-7b-5p (AUC, 0.764 [95%CIs, 0.559 to 0.969]), miR-374a-5p (AUC, 0.799 [95%CIs, 0.619 to 0.978]), miR-93-5p (AUC, 0.750 [95%CIs, 0.536 to 0.964]), miR-186-5p (AUC, 0.889 [95%CIs, 0.726 to 1.000]), miR-151a-3p (AUC, 0.757 [95%CIs, 0.556 to 0.957]) (**Figure 2B**). Sensitivity and specificity of single miRNAs are shown in **Supplementary Table 3**.

To investigate whether there was a miRNA signature with better diagnostic performance, we conducted logistic regression analysis on these 10 miRNAs and obtained a set where all elements (miR-20a-5p, miR-320a-3p, miR-93-5p) were significant. Following that, we calculated the AUC values of all subsets of this set and evaluated their diagnostic performance using ROC. Remarkably, there were two miRNA signatures with AUC values reaching 1.000 in seven subsets, namely Combine 1 (miR-20a-5p + miR-320a-3p + miR-93-5p) and Combine 2 (miR-20a-5p + miR-93-5p) (**Figures 2C, D**). Through logistic regression analysis and the methods previously described (Li et al., 2023), we obtained the formula 1 (diagnostic score (DS) = 16.510 –191.47 × miR-93-5p + 20.508 × miR-320a-3p + 47.873 × miR-20a-5p) for Combine 1 and the formula 2 (DS = 17.688 – 186.101 × miR-93-5p + 59.541 × miR-20a-5p) for Combine 2. Meanwhile, we compared the sensitivity and specificity of the two miRNA signatures and found that they both reached 1.000 (**Figure 2E**). Additionally, calibration curve, residual analysis, and cross-validation were conducted to test the two miRNA signatures by using R, and results showed that both miRNA signatures have excellent diagnostic performance (**Supplementary Figures 4A–D**).

### The miR-20a-5p and miR-93-5p combination showed stronger diagnostic performance in the preliminary validation set

**Figure 3:**
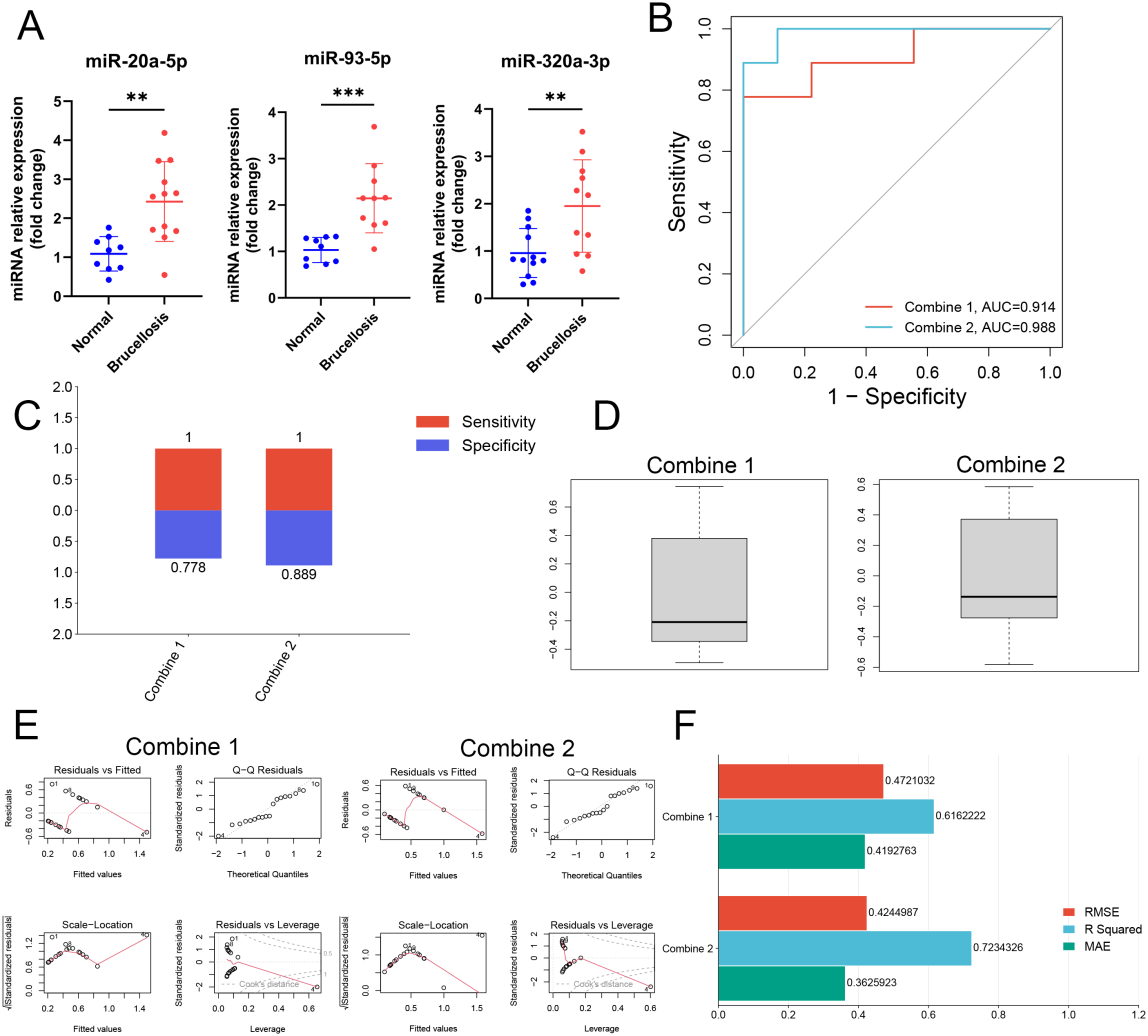
qRT-PCR of miRNAs derived from preliminary validation set, and comparison of the performance of combine 1 and combine 2. **(A)** Expression levels of miR-20a-5p, miR-320a-3p, and miR-93-5p extracted from EVs were examined using unpaired T-test (n=10-12). **(B)** ROC, **(C)** sensitivity and specificity of Combine 1 and Combine 2. **(D)** Box plots are used to reflect the distribution of residual values of Combine 1 and Combine 2. **(E)** Result charts of residual analysis of Combine 1 and Combine 2, including Residuals vs Fitted, Q-Q Residuals, Scale-Location, and Residuals vs Leverage. **(F)** Visualization of cross-validation results of Combine 1 and Combine 2. Data are mean ± SDs; * *p* < 0.05; ** *p* < 0.01; *** *p* < 0.001; **** *p* < 0.0001.

To obtain the best miRNA signature, we conducted qRT-PCR to further detect the expression levels of miR-20a-5p, miR-320a-3p, and miR-93-5p derived from EVs, serum free of EVs (EVs-free), and serum in the preliminary validation set (12 patients with brucellosis and 12 healthy individuals). Compared with that in N-EVs, the expression levels of these three miRNAs were significantly upregulated in B-EVs (**Figure 3A**). Similar to the results of the exploratory set, no significant differences in the expression levels of these three miRNAs were observed between patients with brucellosis and healthy individuals in either EVs-free serum or serum samples (**Supplementary Figures 4E, F**). We used formulas 1 and formulas 2 obtained from the exploratory set to calculate the DS of Combine 1 and Combine 2 in the preliminary validation set, respectively, and used the DSs for subsequent analysis. ROC analysis revealed that the AUC of Combine 2 (AUC, 0.988 [95%CIs, 0.949 to 1.000]) was greater than that of Combine 1 (AUC, 0.914 [95%CIs, 0.776 to 1.000]) (**Figure 3B**). Combine 2 (sensitivity, 1.000; specificity, 0.889) also had better sensitivity and specificity than Combine 1 (sensitivity, 1.000; specificity, 0.778) (**Figure 3C**). Furthermore, residual analysis and cross-validation also showed that Combine 2 is the preferable miRNA signature. (**Figures 3D–F**). Subsequently, we collected samples from patients with *Mycobacterium tuberculosis* infection, a common clinical condition that shares similar clinical symptoms with brucellosis and is easily confused with it, to further evaluate the differential diagnostic performance of these two miRNAs. The results showed that the levels of these two miRNAs were significantly decreased in serum EVs from patients with *M. tuberculosis* infection (**Supplementary Figure 5**). Therefore, these two miRNAs not only effectively distinguish brucellosis patients from healthy individuals but also demonstrate good differential diagnostic performance among diseases with overlapping clinical symptoms (such as *M. tuberculosis* infection), suggesting their potential value as specific diagnostic biomarkers for brucellosis. Therefore, miR-20a-5p and miR-93-5p combination may serve as useful diagnostic biomarkers for brucellosis.

### miR-20a-5p and miR-93-5p showed potential association with brucellosis progression

**Figure 4:**
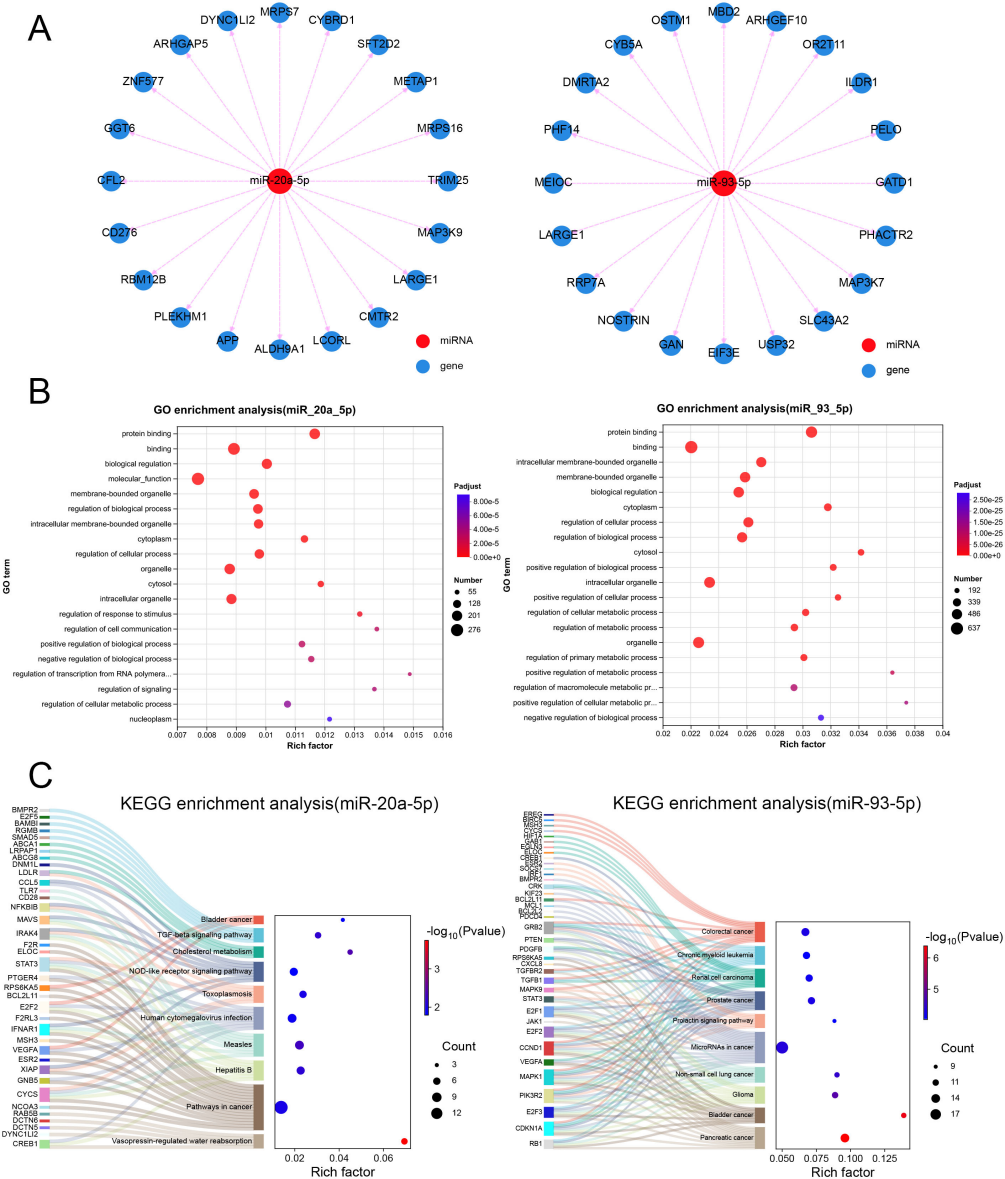
Prediction and functional evaluation of target genes of miRNAs. **(A)** Interaction networks of miRNAs with the top 20 target genes. **(B)** Gene ontology (GO) enrichment analysis and **(C)** Kyoto Encyclopedia of Genes and Genomes (KEGG) pathway for functional evaluation of target genes of miRNAs.

To obtain an overview of the biological functions associated with the two EVs-miRNAs (miR-20a-5p and miR-93-5p), we predicted the target messenger RNAs (mRNAs) for these two miRNAs and presented the top 20 mRNAs with the highest prediction scores through an interaction network diagram (**Figure 4A**). The target mRNAs were used to perform GO enrichment analysis and KEGG pathway analysis. The GO analysis results indicated that the target mRNAs were significantly enriched in multiple aspects of biological processes, including protein binding, regulation of cell communication, transcriptional regulation of RNA polymerase, and regulation of the cellular metabolic process, which play important roles in gene regulation and disease development (**Figure 4B**). In addition, the target mRNAs were significantly enriched in pathogen infection and inflammation-related pathways (**Figure 4C**) (Wang et al., 2024; Wei et al., 2025). Although the miRNAs identified in this study are not exclusively expressed in brucellosis, their combined expression pattern showed promising diagnostic performance, and their opposite expression in tuberculosis suggests potential utility for differential diagnosis. Their involvement in inflammatory pathways may also indicate a role in disease progression. Further validation in larger and more specific sets is needed.

## Discussion

In this study, a comprehensive biomarker discovery program was performed to provide a 2-miRNA diagnostic signature for patients with brucellosis based on miRNAs derived from serum EVs. Specifically, we developed and validated novel diagnostic tools based on the DS of the combination of EVs-miR-20a-5p and EVs-miR-93-5p. This combination showed the best performance for the diagnosis of brucellosis.

To date, there have been three main types of diagnostic methods for brucellosis: culture-based, serological tests, and nucleic acid-amplification assays (Yagupsky et al., 2019). Although a positive result from bacterial culture is the gold standard for diagnosing brucellosis, slow bacterial growth is an inevitable drawback, and there is a risk of misdiagnosis and missed diagnosis owing to insufficient cultivation time (Yilmaz et al., 2016). Because of the significant differences in individual recognition of non-self antigens, immunological processing, and resulting patterns and dynamics of antibody production, serological test results for brucellosis are often considered subjective, indirect, and unreliable diagnostic evidence (Al Dahouk and Nöckler, 2011; Yagupsky et al., 2019). Nucleic acid-amplification assays may be an alternative to conventional microbiological diagnosis for brucellosis. Nowadays, the nucleic acid amplification assays for diagnosing brucellosis are similar to those for other microorganisms, that is, they detect the genomic targets of *Brucella* based on the polymerase chain reaction (PCR) technique. Unfortunately, the total number of existing genomic targets of *Brucella* is still relatively small (García-Yoldi et al., 2006; Bounaadja et al., 2009; Yagupsky et al., 2019). The two-miRNA diagnostic signature for brucellosis developed in this study is based on qRT-PCR of host-derived nucleic acids, and represents the first miRNA signature using serum EVs-miRNAs for the diagnosis of brucellosis. This miRNA signature offers the advantages of nucleic acid-amplification assays as well as provides an important personalized diagnostic basis for brucellosis. Based on the promising diagnostic performance of the host-derived EVs-miRNA panel identified in this study, we propose that future studies combining the detection of host EVs-miRNAs with *Brucella* DNA within EVs may further improve diagnostic accuracy and help reduce missed diagnosis and misdiagnosis of brucellosis.

Circulating miRNAs detected in EVs-free serum are now widely recognized to originate from multiple sources. A substantial proportion of extracellular miRNAs is not encapsulated within vesicles, but instead exists in association with RNA-binding proteins, particularly Argonaute 2 (Ago2), or with lipoproteins such as high-density lipoprotein (HDL) (Arroyo et al., 2011; Vickers et al., 2011). Importantly, EVs-free miRNAs represent a relatively heterogeneous population, reflecting a mixture of passive release during cell death, non-specific secretion, and cellular turnover. This heterogeneity may introduce significant variability and limit their disease specificity, making them less reliable as diagnostic biomarkers. In contrast, EVs-miRNAs are enclosed within lipid bilayers and are generally considered to be actively released through regulated processes, thereby more likely to reflect specific biological events and intercellular communication (Turchinovich et al., 2011; Yáñez-Mó et al., 2015). Given these advantages in stability, specificity, and biological relevance, we chose to focus on EVs-miRNAs rather than EVs-free miRNAs as the basis for our diagnostic model. Therefore, although EVs-free miRNAs constitute an important component of circulating miRNAs, EVs-miRNAs offer superior reliability for biomarker discovery. Consistent with this rationale, our own data showed no significant differences in EVs-free miRNA levels between brucellosis patients and healthy controls, further supporting the choice of EVs-miRNAs as more informative diagnostic markers. miRNAs are selectively loaded, rather than randomly, into EVs. This indicates that EVs are rich in unique repertories of miRNAs, which not only accurately reflect the state of the originating cells, but also their composition (Nabhan et al., 2012; Villarroya-Beltri et al., 2013; Matsumura et al., 2015; Shurtleff et al., 2017). Previous studies have demonstrated the use of EV-miRNAs as diagnostic and prognostic biomarkers for various diseases, including infections and cancer (Driedonks et al., 2020; He et al., 2021; Prattichizzo et al., 2021; Li et al., 2023; Li et al., 2024). In addition, accumulating evidence suggests that EVs derived from *Brucella*-infected cells play important roles in host–pathogen interactions. Proteomic analysis of exosomes from Brucella abortus-infected macrophages revealed that these vesicles carry proteins associated with immune regulation, indicating their involvement in immune evasion and host modulation (Álvarez et al., 2025). EVs released by *Brucella*-infected macrophages have been shown to restrict intracellular *Brucella* survival by driving macrophage polarization toward the M1 phenotype, thereby enhancing host antibacterial immune defense (Wang et al., 2023). miRNAs can regulate the *Brucella*-host interactions, playing an essential role in the pathogenesis and progression of brucellosis (Zheng et al., 2012; Budak et al., 2018). EVs-miRNAs thus serve as feasible diagnostic biomarkers for brucellosis and have potential as therapeutic targets.

Consistently, previous studies have demonstrated that specific miRNAs directly participate in regulating *Brucella* infection. For instance, miR-125b-5p has been shown to suppress *Brucella* abortus intracellular survival by controlling A20 expression, thereby modulating inflammatory signaling pathways (Liu et al., 2016). Furthermore, comprehensive analyses have identified multiple differentially expressed serum miRNAs in humans responding to *Brucella* infection (Zhang et al., 2019), and altered expression of miRNAs such as miR-1238-3p, miR-494, miR-6069, and miR-139-3p has been associated with chronic brucellosis, suggesting that miRNAs are closely related to disease progression and host immune status (Budak et al., 2016a).

We found that among the serum EVs-miRNAs that we identified through studies, the combination of miR-20a-5p and miR-93-5p was identified as the best miRNA diagnostic signature, with high sensitivity and specificity. MiR-20a-5p can balance macrophage inflammatory polarization and regulate the immune response of B cells (Xu et al., 2022; Mao et al., 2024). Some studies had found that miR-20a-5p regulates the immune response by controlling the MAPK and apoptosis signaling pathways. Additionally, it can regulate the immune response during Avian pathogenic *E. coli* infection by targeting transforming growth factor-beta receptor 2 (TGFBR2), thereby inhibiting the production of inflammatory cytokines (Hong et al., 2023; Cao et al., 2024). Previous study showed that miR-93-5p plays a role in regulating innate and adaptive immunity, and mediates the development of sepsis (Dragomir et al., 2023). MiR-93-5p can reduce polarization of M1 macrophages and suppress immune responses by targeting TLR4 and IRF1 mRNA (Liu et al., 2020). Moreover, miR-93-5p can affect the production of ROS, thereby regulating oxidative stress-triggered inflammation (Yang et al., 2021). This may be a potential mechanism by which *Brucella* regulates host immune defense and implements immune escape. However, there is currently no research indicating the differential expression levels and potential functional mechanisms of miR-20a-5p and miR-93-5p in patients with brucellosis. Our study has identified, for the first time, that miR-20a-5p and miR-93-5p have significant differential expression levels in serum-derived EVs between patients with brucellosis and healthy individuals, and have good diagnostic efficacy in diagnosing and distinguishing brucellosis, as verified by our set studies.

The limitations of this study are worth mentioning. The relatively small sample size of both sets underscores the importance of expanding participant recruitment and establishing multicenter collaborations to strengthen the statistical power and clinical applicability of our findings. While the current miRNA diagnostic signature demonstrates promising performance in brucellosis-confirmed cases, its real-world utility requires rigorous validation through prospective studies involving patients with suspected brucellosis, particularly to evaluate diagnostic accuracy in early or atypical presentations. Furthermore, additional validation in larger sample sizes is still necessary.

## Conclusions

Our study revealed significant differences in serum EVs-miRNA profiles between brucellosis patients and healthy controls. A two-miRNA signature (EVs-miR-20a-5p and EVs-miR-93-5p) was identified and validated. These miRNAs were elevated in brucellosis and enriched in inflammatory pathways. ROC analysis showed promising diagnostic performance, though further validation is needed. The main contribution of this study is to highlight the differential expression of EVs-miRNAs in *Brucella* infection and to provide a basis for mechanistic studies.

## Data Availability

The original contributions presented in the study are included in the article/**Supplementary Material**. Further inquiries can be directed to the corresponding authors.
