# Supplementary material for: Extracellular vesicle microRNA signature as a highly accurate diagnostic biomarker for human brucellosis
Source: Front Cell Infect Microbiol. 2026 May 22;16:1806232. doi: 10.3389/fcimb.2026.1806232 (PMC13236866; doi:10.3389/fcimb.2026.1806232)
Supplement: Supplementary file 1 [file Table1.docx]

**Supplementary material**

**Table S1. Primer sequences of the top 12 miRNAs with the highest fold change.**

| **miRNA** | **Primer Sequence** |
| --- | --- |
| hsa-miR-93-5p | CAACAAAGTGCTGTTCGTGC |
| hsa-miR-181b-5p | ATTCATTGCTGTCGGTGGGT |
| hsa-miR-98-5p | GGGTGAGGTAGTAAGTTGTAT |
| hsa-miR-361-5p | TTATCAGAATCTCCAGGGGTA |
| hsa-miR-151a-3p | CTAGACTGAAGCTCCTTGAG |
| hsa-miR-19b-3p | GTGCAAATCCATGCAAAACTG |
| hsa-let-7d-5p | GAGGTAGTAGGTTGCATAGTT |
| hsa-miR-186-5p | CAAAGAATTCTCCTTTTGGGC |
| hsa-miR-374a-5p | CCGCCTTATAATACAACCTGA |
| hsa-let-7b-5p | GAGGTAGTAGGTTGTGTGGT |
| hsa-miR-320a-3p | AAAGCTGGGTTGAGAGGGC |
| hsa-miR-20a-5p | AAGTGCTTATAGTGCAGGTAG |

**Table S2. Numerical results of small RNA sequencing results, including 52 significantly up-regulated miRNAs (*p* < 0.05).**

| **miRNA** | **Fold change** | **p-value** |
| --- | --- | --- |
| hsa-miR-181b-5p | 12.15294118 | 1.46E-07 |
| hsa-miR-98-5p | 12.15294118 | 1.46E-07 |
| hsa-miR-361-5p | 12.15294118 | 1.46E-07 |
| hsa-miR-151a-3p | 12.15294118 | 1.46E-07 |
| hsa-miR-19b-3p | 12.15294118 | 1.46E-07 |
| hsa-let-7d-5p | 12.15294118 | 1.46E-07 |
| hsa-miR-186-5p | 12.15294118 | 1.46E-07 |
| hsa-miR-93-5p | 10.12745098 | 4.15E-13 |
| hsa-miR-374a-5p | 10.12745098 | 3.35E-07 |
| hsa-let-7b-5p | 10.12745098 | 3.35E-07 |
| hsa-miR-320a-3p | 10.12745098 | 3.35E-07 |
| hsa-miR-20a-5p | 10.12745098 | 3.35E-07 |
| hsa-miR-452-5p | 8.680672269 | 7.34E-07 |
| hsa-miR-193b-3p | 8.680672269 | 7.34E-07 |
| hsa-miR-22-3p | 8.680672269 | 7.34E-07 |
| hsa-miR-101-3p | 8.680672269 | 7.34E-07 |
| hsa-let-7d-3p | 8.680672269 | 7.34E-07 |
| hsa-miR-31-5p | 8.680672269 | 7.34E-07 |
| hsa-miR-140-3p | 7.595588235 | 1.54E-06 |
| hsa-miR-181a-5p | 7.595588235 | 1.54E-06 |
| hsa-miR-224-5p | 7.595588235 | 1.54E-06 |
| hsa-miR-365b-3p | 6.751633987 | 3.11E-06 |
| hsa-miR-365a-3p | 6.751633987 | 3.11E-06 |
| hsa-miR-27a-3p | 6.751633987 | 3.11E-06 |
| hsa-miR-221-3p | 6.751633987 | 3.11E-06 |
| hsa-let-7i-5p | 6.076470588 | 1.21E-10 |
| hsa-miR-26b-5p | 6.076470588 | 6.04E-06 |
| hsa-miR-425-5p | 6.076470588 | 6.04E-06 |
| hsa-miR-455-3p | 6.076470588 | 6.04E-06 |
| hsa-miR-17-5p | 6.076470588 | 6.04E-06 |
| hsa-miR-27b-3p | 6.076470588 | 6.04E-06 |
| hsa-miR-23b-3p | 5.524064171 | 1.13E-05 |
| hsa-miR-106b-5p | 5.524064171 | 1.13E-05 |
| hsa-miR-25-3p | 5.283887468 | 7.45E-10 |
| hsa-miR-222-3p | 5.06372549 | 2.05E-05 |
| hsa-miR-125a-5p | 5.06372549 | 2.05E-05 |
| hsa-miR-92b-3p | 4.674208145 | 3.59E-05 |
| hsa-miR-30e-5p | 4.340336134 | 6.11E-05 |
| hsa-miR-99b-5p | 4.050980392 | 0.000101 |
| hsa-miR-15b-5p | 3.797794118 | 0.000164 |
| hsa-miR-148b-3p | 3.574394464 | 0.000258 |
| hsa-miR-29a-3p | 3.574394464 | 0.000258 |
| hsa-let-7g-5p | 3.198142415 | 0.000602 |
| hsa-miR-99a-5p | 3.038235294 | 0.000893 |
| hsa-miR-183-5p | 3.038235294 | 0.000893 |
| hsa-miR-125b-5p | 2.893557423 | 0.001300 |
| hsa-miR-16-5p | 2.762032086 | 0.001859 |
| hsa-miR-191-5p | 2.762032086 | 0.001859 |
| hsa-miR-199b-3p | 2.430588235 | 0.004949 |
| hsa-miR-199a-3p | 2.430588235 | 0.004949 |
| hsa-miR-423-3p | 2.250544662 | 0.000184 |
| hsa-miR-24-3p | 2.170168067 | 0.011615 |

**Table S3. Area under the curve (AUC), sensitivity, and specificity of single miRNA.**

| **miRNA** | **Area** | **Sig.** | **Lower Bound** | **Upper Bound** | **Sensitivity** | **Specificity** |
| --- | --- | --- | --- | --- | --- | --- |
| hsa-miR-93-5p | 0.750 | 0.038 | 0.536 | 0.964 | 0.583 | 1.000 |
| hsa-miR-98-5p | 0.910 | 0.001 | 0.788 | 1.000 | 0.833 | 0.917 |
| hsa-miR-361-5p | 0.917 | 0.001 | 0.760 | 1.000 | 0.917 | 1.000 |
| hsa-miR-151a-3p | 0.757 | 0.033 | 0.556 | 0.957 | 0.583 | 1.000 |
| hsa-let-7d-5p | 0.903 | 0.001 | 0.761 | 1.000 | 0.917 | 0.917 |
| hsa-miR-186-5p | 0.889 | 0.001 | 0.726 | 1.000 | 0.833 | 1.000 |
| hsa-miR-374a-5p | 0.799 | 0.013 | 0.619 | 0.978 | 0.833 | 0.667 |
| hsa-let-7b-5p | 0.764 | 0.028 | 0.559 | 0.969 | 0.667 | 0.917 |
| hsa-miR-320a-3p | 0.715 | 0.073 | 0.501 | 0.929 | 0.583 | 0.833 |
| hsa-miR-20a-5p | 0.903 | 0.001 | 0.745 | 1.000 | 0.833 | 1.000 |


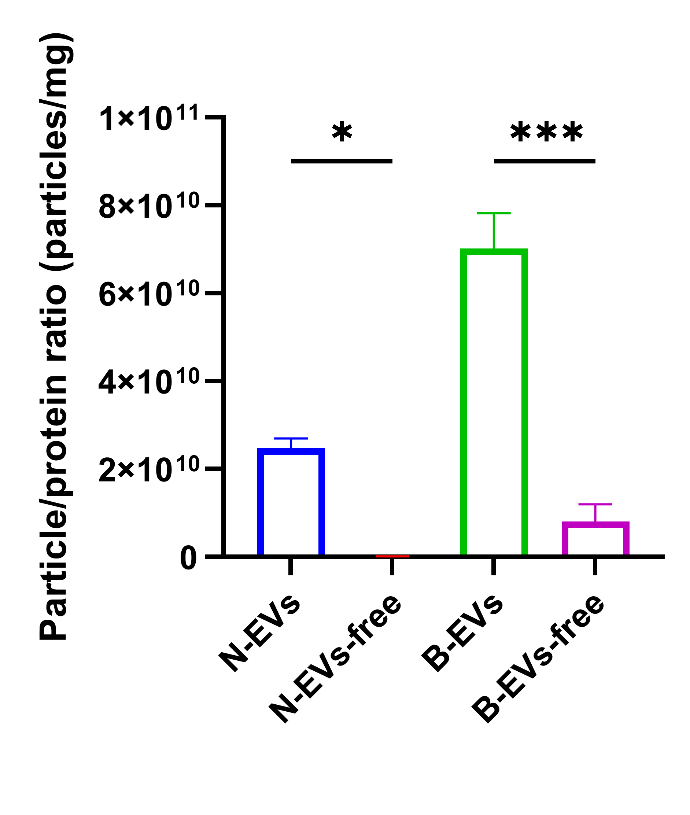


**Fig. S1. The particle-to-protein ratio (particles per mL divided by mg per mL) was determined for both serum-derived EVs and the corresponding EV-free obtained from healthy subjects and brucellosis patients.**


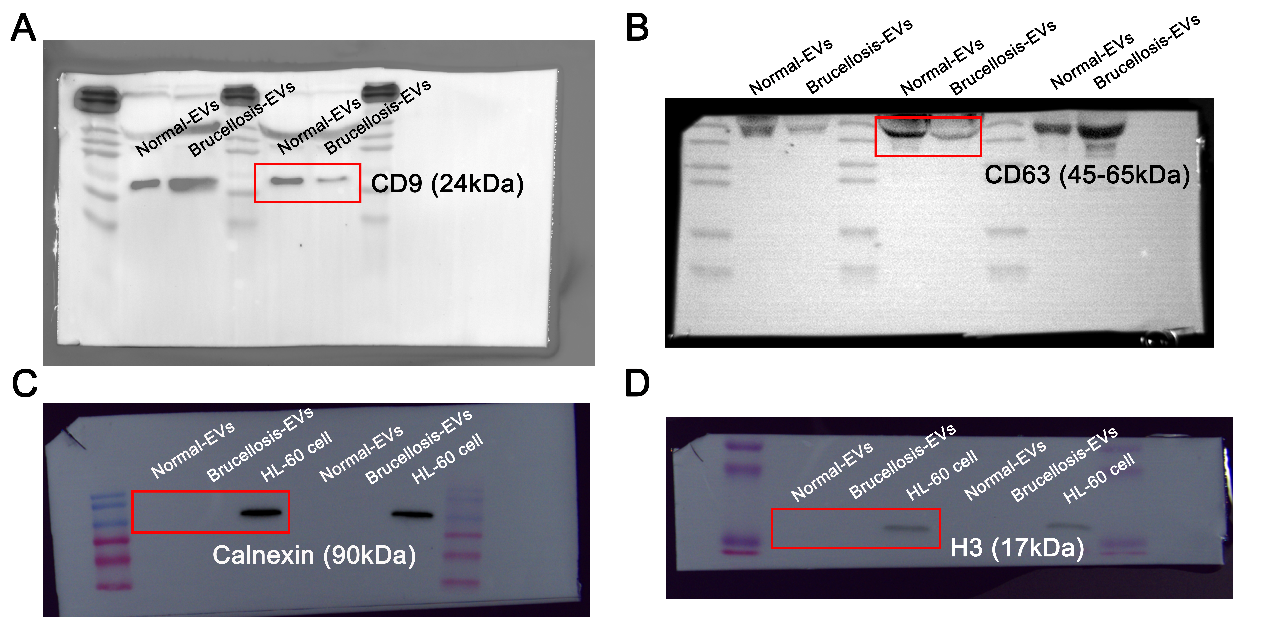


**Fig. S2. The full uncropped Gels and Blots images.** The original Gels results of Western Blot of CD9 (A), CD63 (B), calnexin (C) and H3 (D).


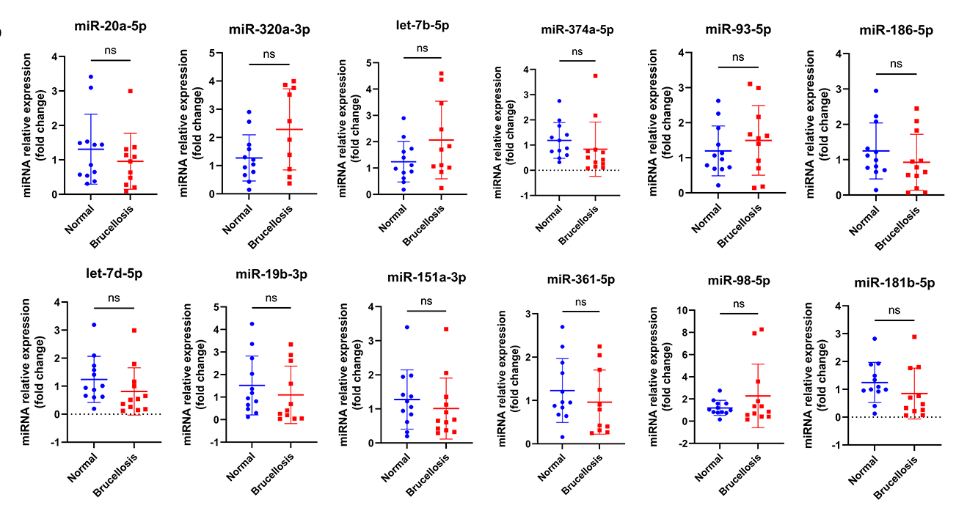


**Fig. S3. Visualization of fold change of miRNAs derived from extracellular vesicles (EVs), and qRT-PCR of miRNAs derived from exploratory set.** Expression levels of miR-20a-5p, miR-320a-3p, let-7b-5p, miR-374a-5p, miR-93-5p, miR-186-5p, let-7d-5p, miR-19b-3p, miR-151a-3p, miR-361-5p, miR-98-5p, miR-181b-5p extracted from serum (n=10-12). Data are mean ± SDs; * *p* < 0.05; ** *p* < 0.01; *** *p* < 0.001; **** *p* < 0.0001.


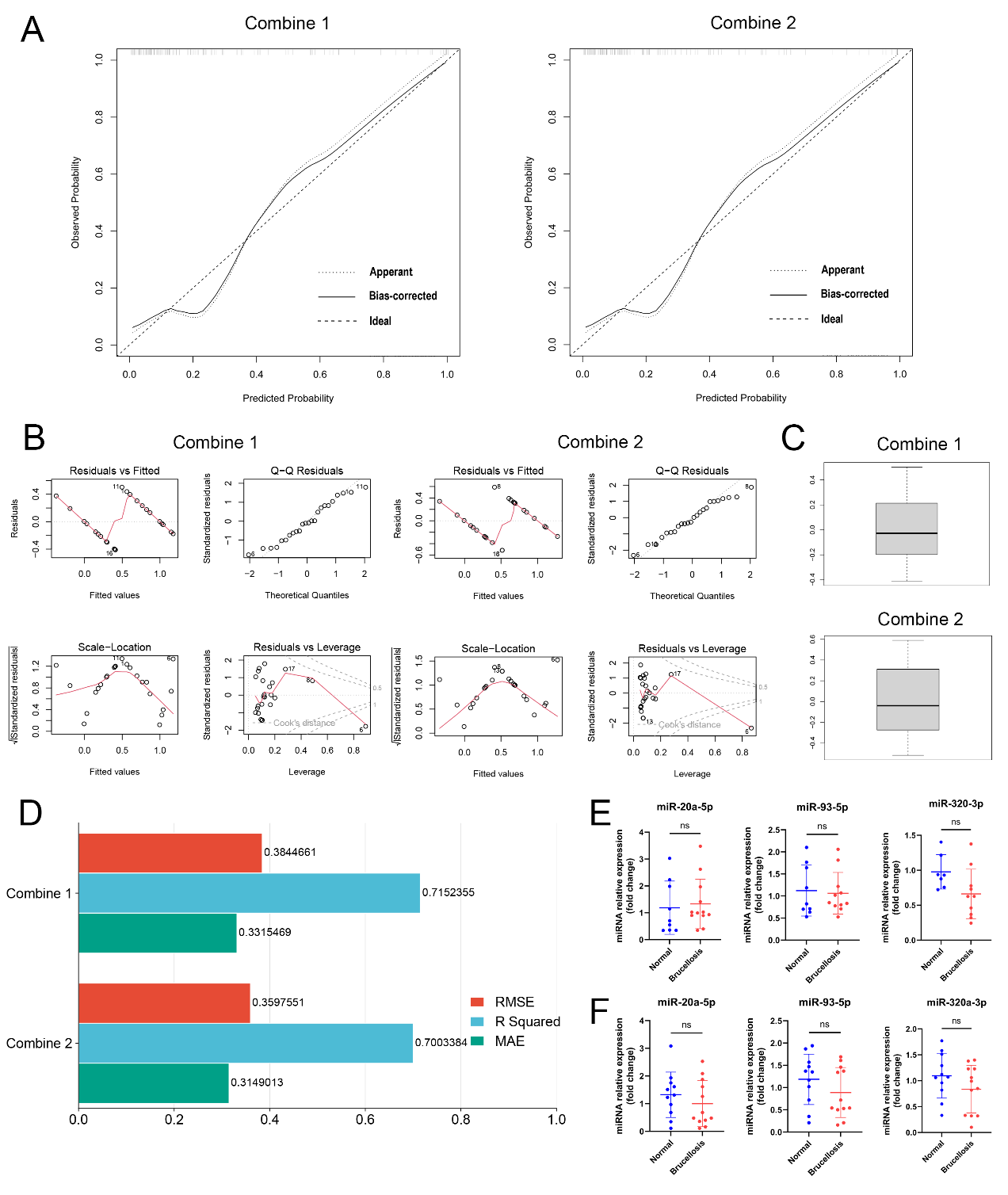


**Fig. S4. Bioinformatics analysis for comparing miRNA signatures and qRT-PCR of miRNAs derived from preliminary validation set.** (A) Calibration curve of Combine 1 (miR-20a-5p + miR-320a-3p + miR-93-5p) and Combine 2 (miR-20a-5p + miR-93-5p). (B) Result charts of residual analysis of Combine 1 and Combine 2, including Residuals vs Fitted, Q-Q Residuals, Scale-Location, and Residuals vs Leverage. (C) Box plots are used to reflect the distribution of residual values of Combine 1 and Combine 2. (D) Visualization of cross-validation results of Combine 1 and Combine 2. Expression levels of miR-20a-5p, miR-320a-3p, and miR-93-5p extracted from (E) EVs-free and (F) serum (n=10-12). Data are mean ± SDs; * *p* < 0.05; ** *p* < 0.01; *** *p* < 0.001; **** *p* < 0.0001.


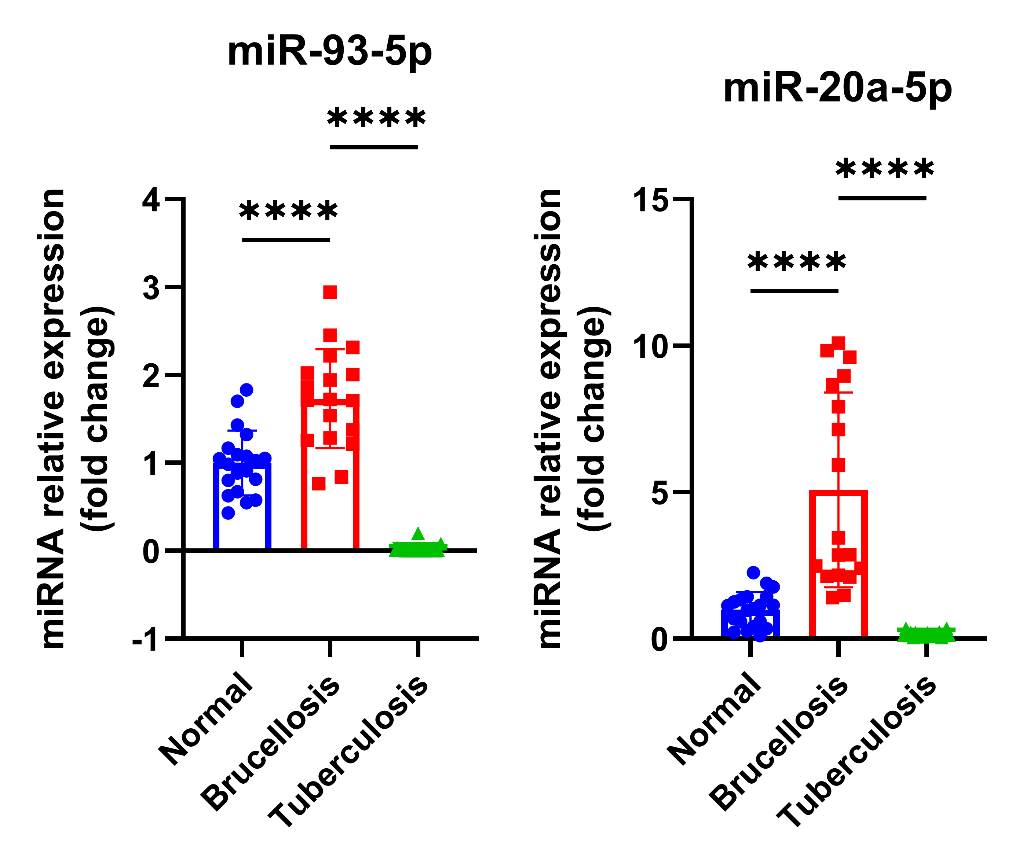


**Fig. S5. Expression levels of miR-20a-5p and miR-93-5p extracted from serum EVs in healthy individuals, brucellosis and tuberculosis (n=18-20).** Data are mean ± SDs; * *p* < 0.05; ** *p* < 0.01; *** *p* < 0.001; **** *p* < 0.0001.
